# Effectiveness of traditional herbal Kampo medicine Goreisan on chronic subdural hematoma recurrence: a meta-analysis

**DOI:** 10.3389/fphar.2024.1412190

**Published:** 2024-07-15

**Authors:** Zhenyu Yang, Yuecheng Zeng, Jinyang Hu, Xin Huang, Haiquan Zhang, Yang Liu

**Affiliations:** ^1^ Department of Neurosurgery, Xiangyang Central Hospital, Affiliated Hospital of Hubei University of Arts and Science, Xiangyang, China; ^2^ Department of Neurosurgery, The First College of Clinical Medical Science, Three Gorges University and Yichang Central People’s Hospital, Yichang, Hubei, China

**Keywords:** Goreisan, traditional herbal Kampo medicine, chronic subdural hematoma, recurrence, meta-analysis

## Abstract

**Objectives:** Our objective was to compare the effectiveness of Traditional Chinese herbal Kampo medicine Goreisan in improving recurrence in patients with chronic subdural hematoma (CSDH).

**Methods:** Eligible randomized controlled trials prospective trials, and retrospective cohort studies were systematically identified through searches of PubMed, Cochrane Library, and CNKI from inception to March 2024. Following the application of predetermined inclusion and exclusion criteria to screen the available studies, main outcome measures were rigorously extracted. RevMan v5.4 software was utilized to evaluate the overall recurrence rate, employing a random-effects model to calculate pooled odds ratios with the Mantel-Haenszel estimation method. Inter-study heterogeneity was assessed using the Cochran Q (Chi-square) test and I2 statistics. Funnel plots were used to evaluate publication bias.

**Results:** Among the 48 articles initially screened for citation, eight were ultimately selected for inclusion in the study. The results of our network meta-analysis indicate that patients with newly diagnosed Chronic subdural hematoma experienced a significantly reduced recurrence rate when treated with Goreisan compared to standard neurosurgical treatment (OR: 0.72; 95% CI 0.61–0.86; *p* = 0.00003). There was no statistically significant difference in the incidence rates of complications, including general fatigue, allergic reactions, hepatic dysfunction, and interstitial pneumonia (OR: 7.21; 95% CI 0.37–141.29; *p* = 0.19).

**Conclusion:** Traditional medicine Goreisan was effective in reducing CDSH recurrence rates. For clinical treatment, it provides a high level of evidence-based medicine. It is also necessary to conduct multicenter randomized controlled trials with dose adjustments to determine whether Goreisan interventions improve neurological function or prognosis.

## Introduction

Chronic subdural hematoma (CSDH) is a common neurological disease, It is reported that 20.6 out of every 100,000 people suffer from chronic diseases annually (Yu et al., 2022). The aging global population is anticipated to contribute to a rise in the prevalence of CSDH cases (Balser et al., 2015). In elderly patients, the presence of enlarged subdural spaces is often linked to the use of oral anticoagulants and cerebral atrophy. CSDH typically occurs following minor trauma, leading to the accumulation of blood between the dura and arachnoid membranes (Yasunaga, 2015).

The burr-hole craniotomy is the standard treatment for CSDH, although studies indicate that recurrence rates can range from 4% to 30% and that it is associated with a poor outcome (Hutchinson et al., 2024). Prevailing theories implicating angiogenesis, inflammation and hyperfibrinolysis are the mechanisms behind reoccurrence (Edlmann et al., 2017; Wu et al., 2023). Based on these theories, Inflammation, angiogenesis and hyperfibrinolysis have been targeted with treatments (Huang et al., 2020; Desir et al., 2023). Nonetheless, recurrence rates remain disconcertingly high, necessitating the discovery of novel treatment targets.

Currently, western medicine dominates drug research for chronic subdural hematomas, whereas traditional medicine is rarely explored. As a traditional herbal Kampo medicine, Goreisan consists of five herbs (Alismatis rhizoma, Atractylodis rhizoma, Polyporus, Poria, and Cinnamomi cortex) (Goto et al., 2018), It has been recently reported that Goreisan acts on aquaporins (AQPs), specifically AQP1/4, and can be used to treat asymptomatic CSDH and prevent its recurrence after surgery (Yano et al., 2017; Harada et al., 2020). However, most of the previous clinical studies on Goreisan’s use to treat CSDH were published in Japanese journals and were small case series. It is therefore controversial whether Goreisan is effective in treating CSDH. And a high level of evidence-based medicine is urgently needed.

Until now, no conventional meta-analysis has investigated the effectiveness of Goreisan as a treatment for CSDH recurrence. This is the first meta-analysis that shows Goreisan’s effectiveness as a treatment for CSDH recurrence.

## Materials and methods

### Meta-analysis

PubMed, Cochrane libraries, and CNKI provide broad access to literature, regardless of year or language, when conducting reviews. A combination of Medical Subject Headings (MeSH) and search terms were used with Boolean logical operators, such as “Chronic subdural hematoma,” “Goreisan,” “Prospective cohort studies,” “Randomized controlled trials,” “Retrospective cohort studies,” and other relevant synonyms.

### Selection criteria

We evaluated all eligible citations and excluded those that failed to meet inclusion criteria or were repeated. We read the full text carefully to further evaluate the article’s relevance. Further exploration of relevant research is conducted by analyzing references in the included articles. All references are processed in Endnote X9 (Research Soft, Philadelphia, United States).

### Inclusion and exclusion criteria

Inclusion criteria included: 1) Chronic Subdural Hematoma was diagnosed by all enrolled patients; 2) Comparative studies should be randomized controlled trials or prospective studies or retrospective; 3) Each study should include at least 30 patients. 4) report key outcome indicators. Specifically, patients with Chronic Subdural Hematoma and those under the age of 18 were excluded. Recurrence rate was the primary outcome measure for patients with Chronic Subdural Hematoma. A secondary outcome was complications associated with the Chinese Traditional medicine Goreisan. In the study, recurrence was defined as a new symptomatic Chronic Subdural Hematoma occurring during the study period.

### Data extraction and quality assessment

A study was carried out by three authors (Zhenyu Yang, Yuecheng Zen, and Jinyang Hu) independently to extract and summarize data eligible for inclusion and exclusion standards. Demographic characteristics and data were analyzed for all articles that were included in the review. To create baseline data for this study, we extracted relevant information such as the title of the study, the author, the year of publication, the country, region, and basic characteristics. Review Manager (Version 5.4), a tool for evaluating bias in included studies, was used to assess study quality.

### Statistical analyses

The overall recurrence rate was evaluated using RevMan v5.4. Pooled odds ratios were assessed using a random-effects model and the Mantel-Haenszel estimation method. To evaluate the heterogeneity between the studies, Cochran’s Q test (Chi-square) was used, as were I2 statistics. An I2 value of more than 50% was considered moderate heterogeneity, and significance was determined using 95% confidence intervals or *p* < 0.05 (Higgins et al., 2003).

## Results

### Patient characteristics and study identification

**FIGURE 1:**
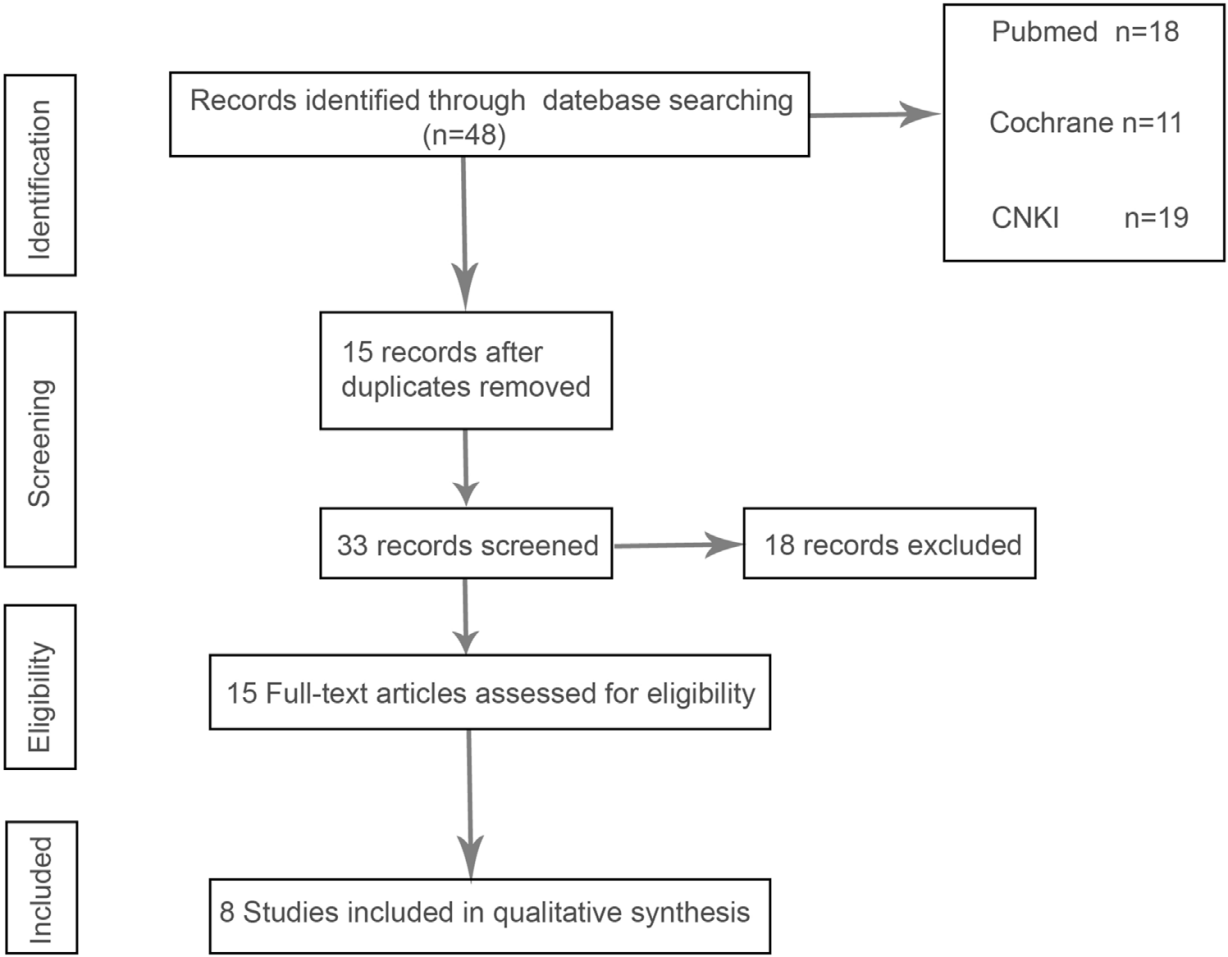
PRISMA flow chart.

**FIGURE 2:**
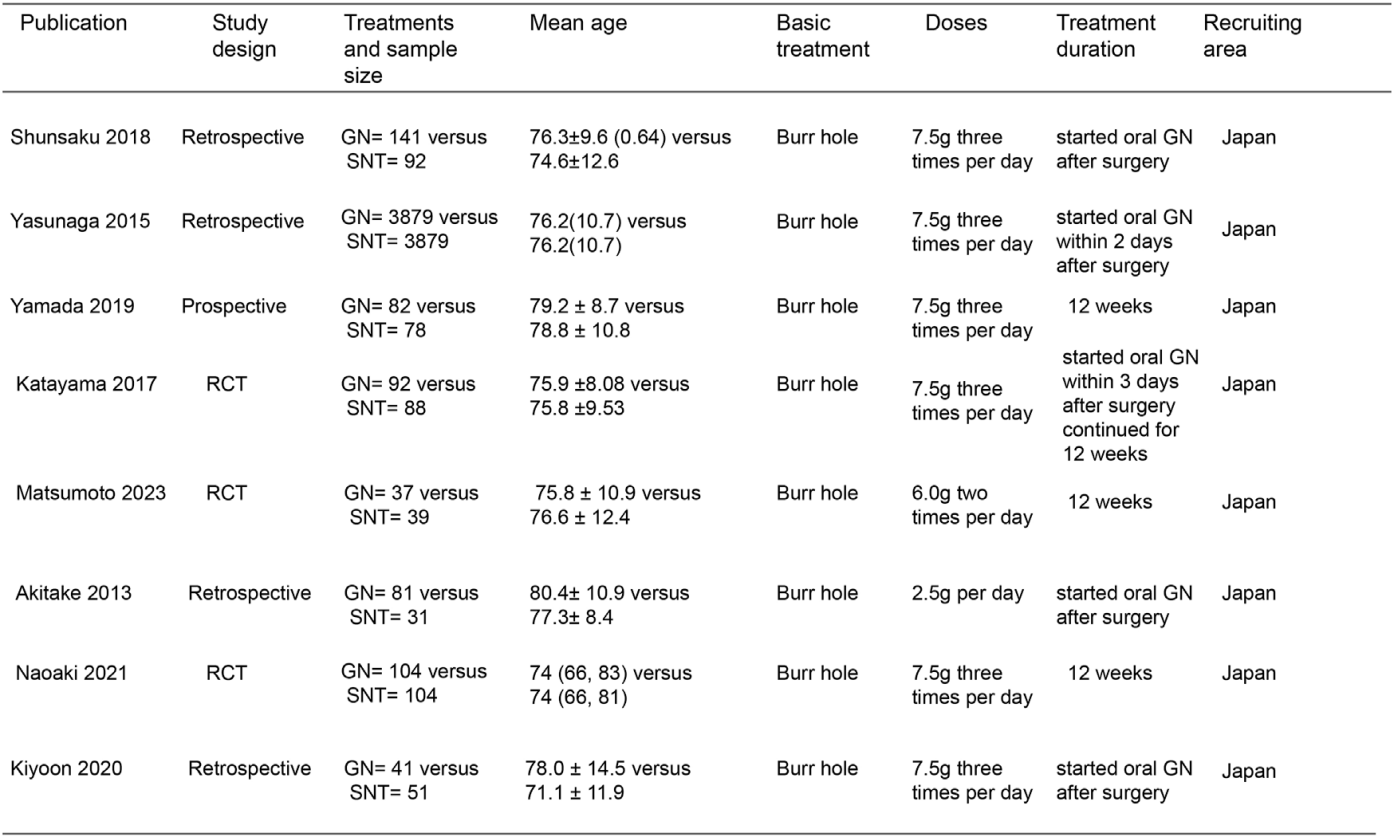
Characteristics of included studies. Note: GN, Goreisan, RCT, randomized controlled study, SNT, standard neurosurgical treatment.

**FIGURE 3:**
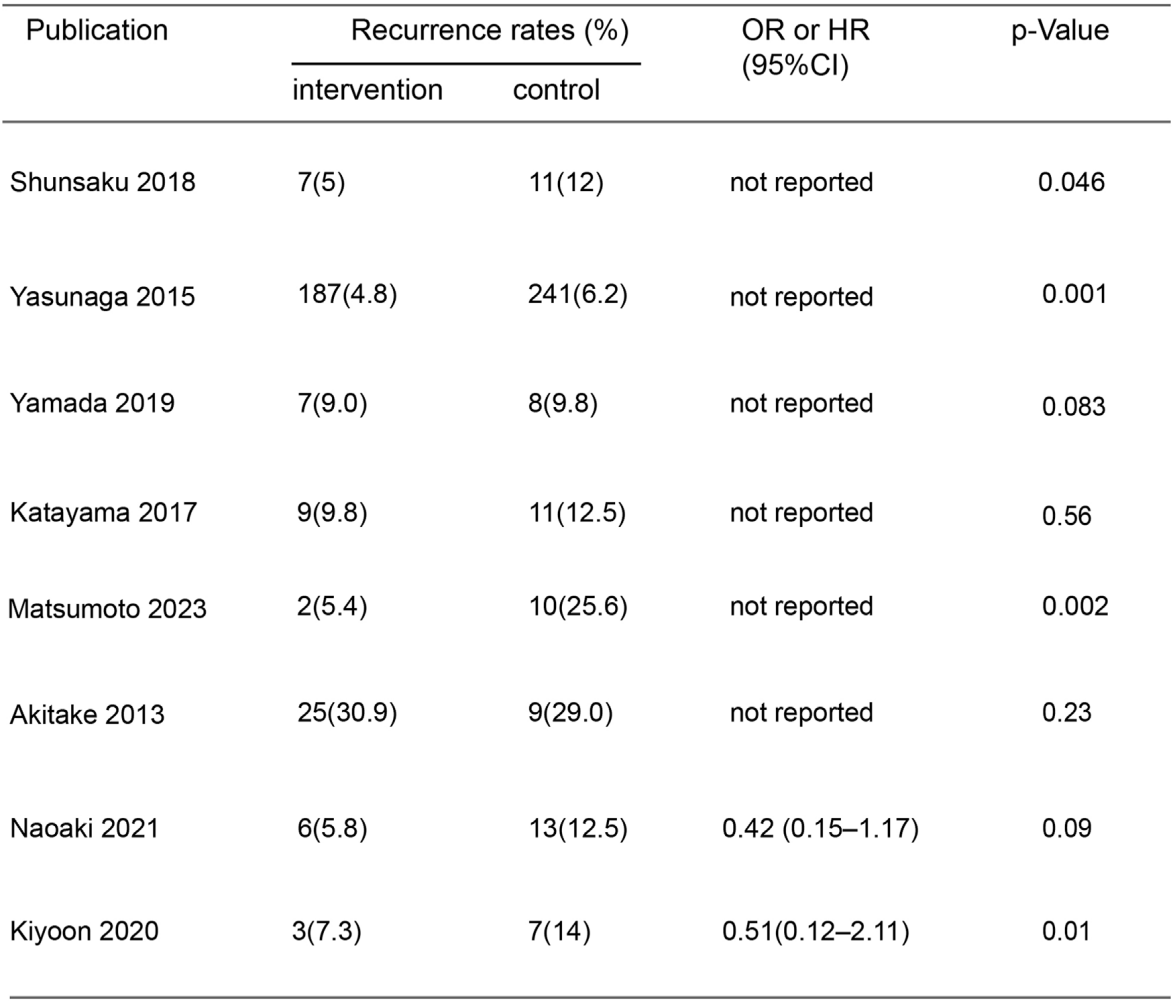
Recurrence rates included in the study.

In the initial screening of 48 pieces of literature, Afterfull-text examination of the remaining articles, 15 articles were included, as seven articles were not RCTs, prospective or retrospective studies, eight were ultimately included after further screening. A flow chart illustrating the process of document selection is shown in Figure 1. The studies included in this study were published from 2013 to 2024. In Figure 2, we summarize the main characteristics and pharmacological interventions of the eight trials that were included. The patients in each study had CSDH. Three articles were RCTs, and one article was prospective studies, four articles were retrospective. Treatment time varied from 1 to 12 weeks, all patients included in the literature underwent trepanation and drainage of subdural hematoma. A summary of the main data from the included trials is presented in Figure 3. Overall, all trials reported recurrence rates; in the intervention group, the rate was 9.75% (range: 5%–30.9%) and in the control group, it was 15.2% (range: 6.2%–29%).

### Risk of bias quality assessment

**FIGURE 4:**
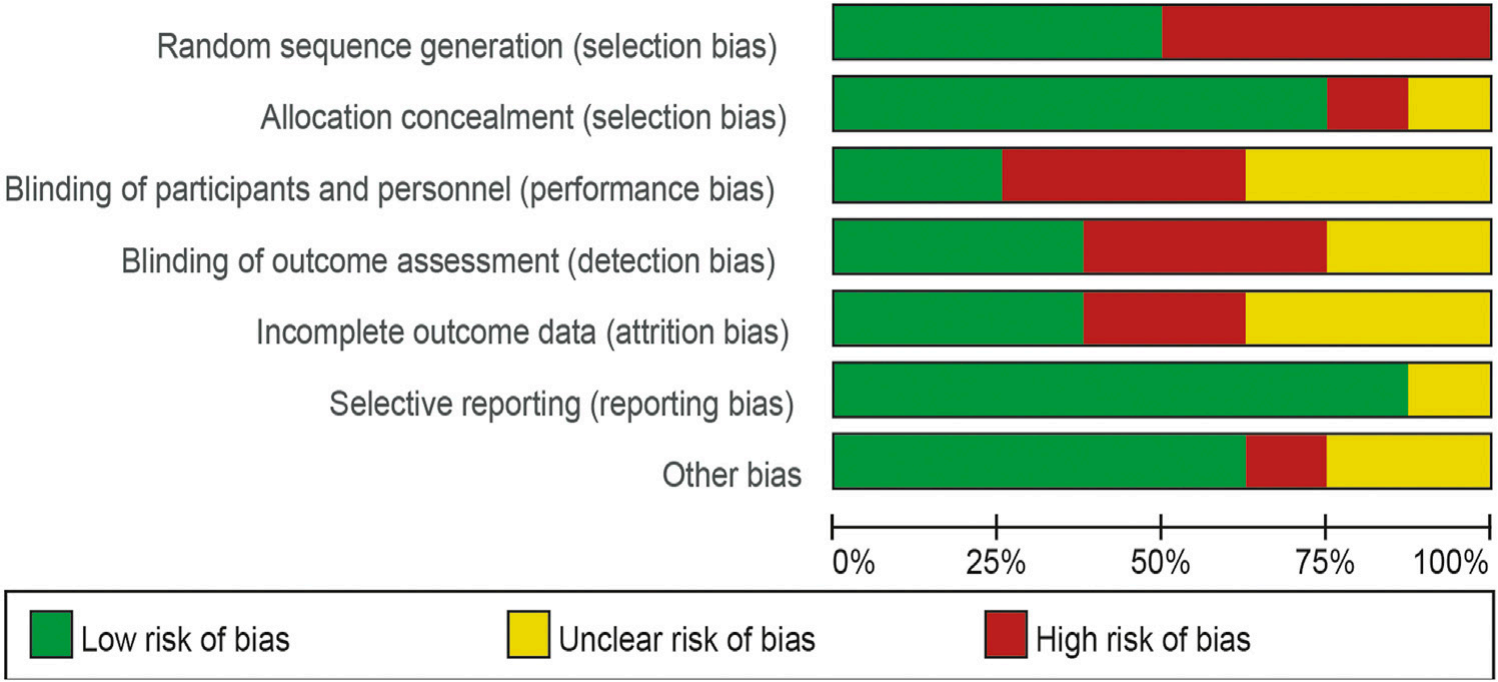
Risk of bias graph: the judgments about each risk of bias item are presented as percentages across all included studies.

**FIGURE 5:**
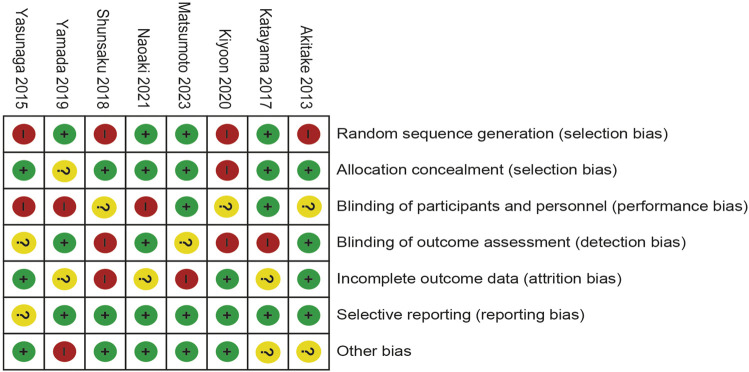
Risk of bias summary: the judgments about each risk of bias item for each included study.

Four of the eight trials included were described in detail as Random sequence generation with low risk. In two studies, blinding of outcomes assessment resulted in unclear risk, which could have led to detection bias. Incomplete outcome data led to five studies being rated as high risk or unclear risk. Figures 4, 5 summarize bias at the individual and population levels.

### Details of the surgical procedure

This paper provides a comprehensive overview of the methodology involved in performing trepanation and drainage for chronic subdural hematoma. The selection of anesthesia is a crucial initial step following the decision to proceed with surgery. Following preoperative imaging analysis, the scalp and subcutaneous tissue are incised at the thickest point of the hematoma, with subsequent drilling of the dura. Post-drilling, a drainage tube is promptly inserted and secured with subcutaneous sutures. The hematoma cavity was irrigated with warm saline solution until the effluent was predominantly cleared, followed by the attachment of the external drainage bag to the drainage tube (Hwang et al., 2022; Uno, 2023).

### Meta-analysis for recurrence rate and secondary outcomes

**FIGURE 6:**
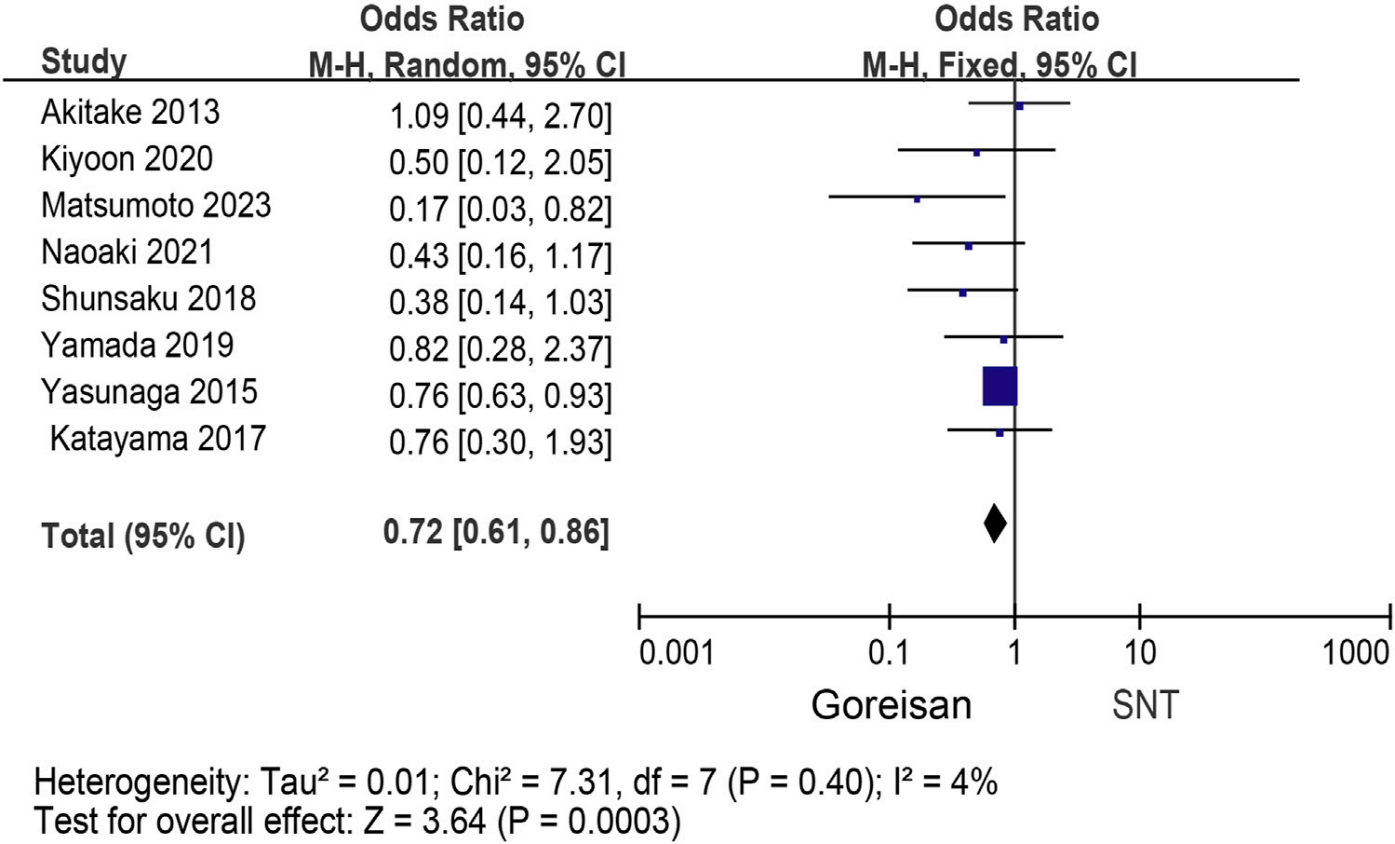
The efficacy of the experimental group was compared with that of the control group.

**FIGURE 7:**
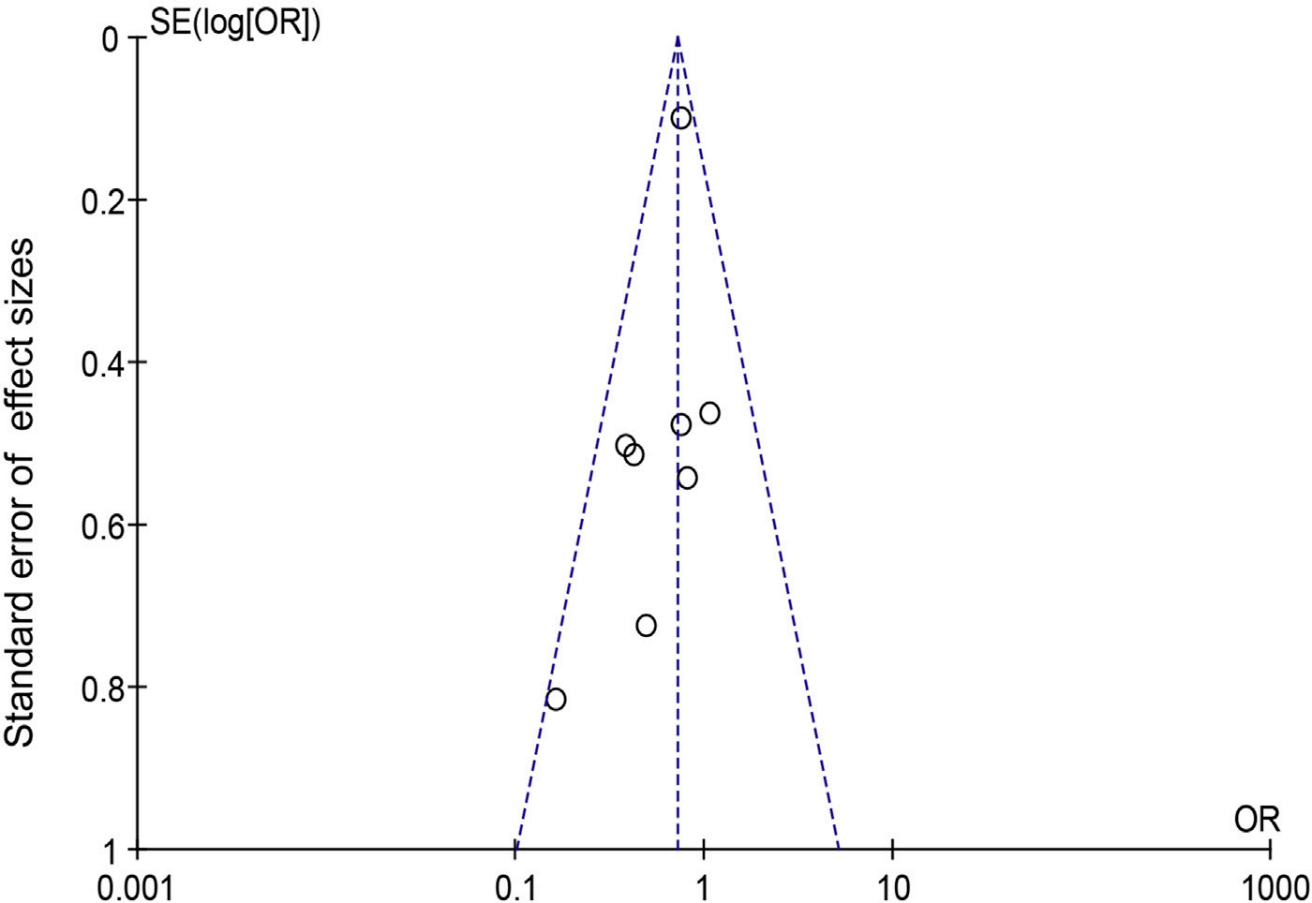
Funnel plot for detecting and displaying system heterogeneity.

**FIGURE 8:**
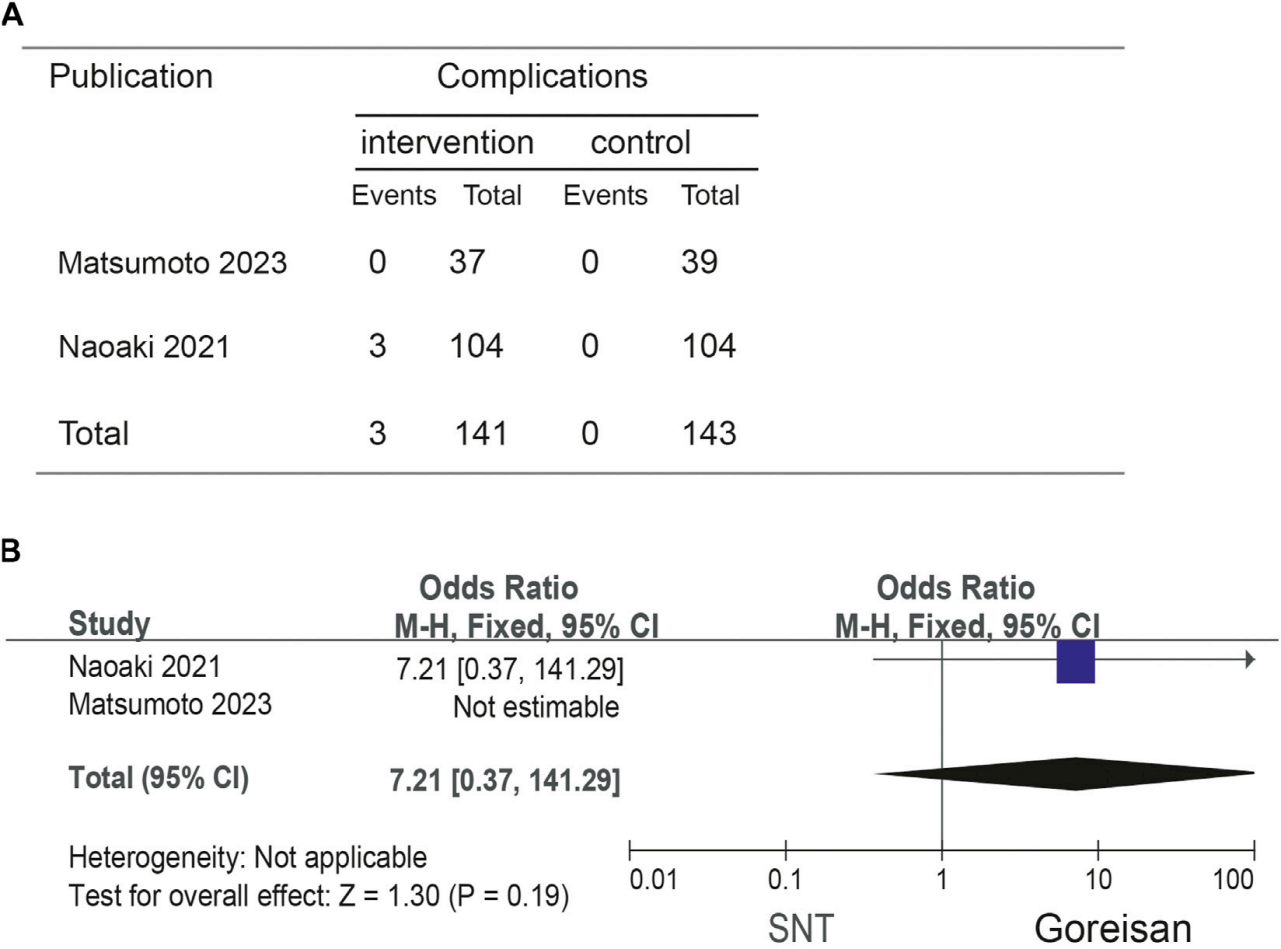
Comparison of complications in the Goreisan Group vs. the Control Group. **(A)** complications results. **(B)** Forest plot of complications.

When Goreisan was used in conjunction with standard neurosurgical treatment to treat newly diagnosed chronic subdural hematomas, the recurrence rate was significantly lower than with standard neurosurgical treatment alone (OR: 0.72; 95% CI 0.61–0.86; *p* = 0.00003). A low level of heterogeneity was found among studies (*I* ^2^ = 7.31%, *p* = 0.40) shown in Figure 6. Inverted funnel plots reveal little asymmetric scattering points, indicating little publication bias shown in Figure 7. Complications (general fatigue, allergic reaction, hepatic dysfunction, and interstitial pneumonia) rates were not significantly different (OR: 7.21; 95% CI 0.37–141.29; *p* = 0.19) Figure 8.

## Discussion

Globally, CSDH incidence and societal impact are predicted to increase as the population ages and anticoagulants become more prevalent, posing an increasing public health threat (Kolias et al., 2014). There is an ongoing debate about the definitive management of chronic subdural hematomas. Although surgical intervention remains the only established treatment option, it is associated with substantial recurrence and mortality rates, estimated at 5%–33%, especially among older and frail patients, increasing morbidity and mortality as well as prolonging hospital stays (Matsumoto et al., 2024).

Numerous studies have indicated that the presence of inflammatory factors and chemokines plays a major role in the development of CSDH and enlargement of the hematoma (de Oliveira et al., 2022; Fan et al., 2022). There is also evidence that high levels of VEGF enhance microangiogenesis and vascular permeability (Edlmann et al., 2022; Petrov et al., 2022). Additionally, the activation of plasminase in CSDH hematomas leads to a significant increase in thrombosis regulatory protein and high fibrinolysis, which promotes blood vessel leakage leading to CSDH progression (Katano et al., 2006; Matsubara et al., 2023). As a result of the understanding that inflammation, angiogenesis, and hyperfibrinolysis play a role in the development of CSDH, there have been several adjunctive therapies used to help reduce recurrence rates, including steroids, angiotensin-converting enzyme inhibitors, tranexamic acid, etizolam, and atorvastatin (Jiang et al., 2018; Tamura et al., 2021). Despite this, there is no consensus on the best adjuvant treatment, and the recurrence rate is still high, necessitating the discovery of novel treatment targets.

AQPs exist in cell membranes and play a key role in maintaining homeostasis and cell migration (Castle, 2005), considered to be a factor affecting the growth of CSDH (Goto et al., 2018), especially AQP1 and AQP4 (Basaldella et al., 2010; Yano et al., 2017). The possible factor leading to the recurrence of CSDH is AQP1 which affects the angiogenesis of the outer membrane (Basaldella et al., 2010). In the central nervous system, aquaporin-4 plays a critical role in increasing membrane permeability to water, which expressed in the outer membrane, especially in areas typically invaded by inflammatory cells, leads to hematoma formation and expansion (Okamura et al., 2013). Several studies have shown that Goreisan could inhibit AQP1 and AQP4 expression and function (Yasunaga, 2015; Yamada and Natori, 2020). This is also a potential mechanism by which Goreisan can reduce the recurrence rate of CDSH. However, the efficacy of Goreisan for CSDH is subject to some controversy in the real world (Okamura et al., 2013; Goto et al., 2018; Katayama et al., 2018; Harada et al., 2020; Yamada and Natori, 2020; Fujisawa et al., 2021; Matsumoto et al., 2024). So, there is an urgent need for high-quality evidence-based medical evidence.

No previous meta-analysis has examined Goreisan’s effect on recurrences of CSDH. Our results suggest that Goreisan can significantly reduce the recurrence rate of CDSH (OR: 0.72; 95% CI 0.61–0.86; *p* = 0.00003), and improve the quality life of patients. Complication (general fatigue, allergic reaction, hepatic dysfunction, and interstitial pneumonia) rates were not significantly different (OR: 7.21; 95% CI 0.37–141.29; *p* = 0.19). However, there are some drawbacks to our study. A major limitation of our study is the lack of randomized controlled trials or prospective studies on Goreisan interventions, and all included studies were in Japanese populations, limiting the evidence on their efficacy and generalisability. In addition, some trials were of low quality, which could potentially undermine the validity of our analysis. It will still be necessary to conduct multicenter, randomized controlled trials to evaluate Goreisan on its own or in combination with other interventions in order to determine if it improves neurological function or prognosis in the future.

## Conclusion

As a result of our findings, Traditional medicine Goreisan was effective in reducing CDSH recurrence rates. For clinical treatment, it provides a high level of evidence-based medicine. It is also necessary to conduct multicenter randomized controlled trials with dose adjustments to determine whether Goreisan interventions improve neurological function or prognosis.

## Data Availability

The original contributions presented in the study are included in the article/Supplementary Material, further inquiries can be directed to the corresponding authors.
